# Cardiac magnetic resonance imaging in today's economic climate; a cost effectiveness analysis

**DOI:** 10.1186/1532-429X-13-S1-M12

**Published:** 2011-02-02

**Authors:** Vinayak A Hegde, J Ronald Mikolich, Mark Doyle, Vikas K Rathi, June A Yamrozik, Ronald B Williams, Robert Biederman

**Affiliations:** 1 Allegheny General Hospital, Pittsburgh, PA, USA; 2 Bon Secours Heart and Vascular Institute, Richmond, VA, USA

## Introduction

In the face of the current health care crisis, significant concerns have been raised about the independent clinical utilities of various cardiac imaging modalities. Particularly, Cardiac Magnetic Resonance (CMR) has been labeled “an expensive pretty picture” by some clinicians as compared to other diagnostic modalities.

## Purpose

### Aim

We hypothesized that results of CMR would independently impact patient management in a cost effective manner.

### Study design

Observational study.

## Methods

We retrospectively reviewed charts of 361 patients (pts) undergoing CMR exams for a variety of indications at two centers in Western Pennsylvania. While Center 1 was an academic center, Center 2 was a private community hospital. Patient outcomes were assessed and the costs of ordering CMR were compared against the benefits to see if CMR indeed resulted in health care savings.

## Results

**Figure 1:**
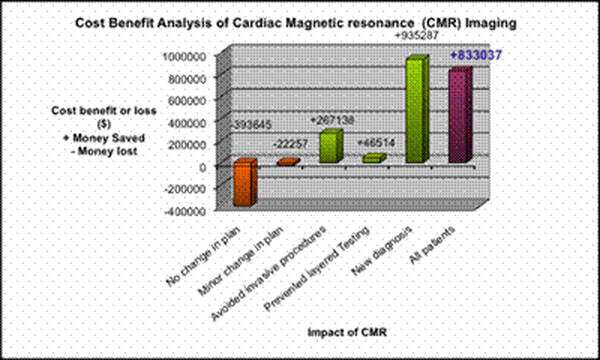


Of the 361 studies, a significant impact was observed in 256 (71%) pts based on CMR results. Of these, 69 (27%) pts received a new diagnosis. Additionally, CMR results avoided invasive procedures in 38 pts and prevented layered testing in 26 pts. Translating these results in to cost benefit analysis, there was a total benefit of $1248,939 and a total loss of $ 415,902, indicating net savings of $ 833,037 for the study group as a whole (see Figure 1). The most savings were observed in pts who received a new diagnosis such as tumor not thrombus, sarcoid not ARVD or benign mass not cancer, and the least, in the routine follow up of clinically stable patients with vascular pathologies such as aneurysms, dissections or pulmonary vein imaging post ablation for atrial fibrillation.

## Conclusions

CMR, when appropriately ordered, independently contributes to patient management, and triggers a major impact on therapeutic decisions in a cost effective manner. As an autonomous diagnostic modality, CMR prevents layered testing and serves a gate keeping function, highlighting the prudent use of cost effective technology.

